# Supplementary material for: Fast Assembly of Metal Organic Framework UiO-66 in Acid-Base Tunable Deep Eutectic Solvent for the Acetalization of Benzaldehyde and Methanol
Source: Molecules. 2022 Oct 25;27(21):7246. doi: 10.3390/molecules27217246 (PMC9657751; doi:10.3390/molecules27217246)
Supplement: Supplementary file 1 [file molecules-27-07246-s001.zip › molecules-1967910-supplementary.pdf]

Supporting Information

Fast Assembly of Metal Organic Framework UiO-66 in Acid-Base Tunable Deep Eutectic Solvent for the Acetalization of Benzaldehyde and Methanol

Lifang Chen *, Xiangzhu Ye, Ting Zhang, Hao Qin, Hongye Cheng and Zhiwen Qi *

State Key Laboratory of Chemical Engineering, School of Chemical Engineering,

East China University of Science and Technology, Shanghai, 200237, P.R. China.

*E-mail: lchen@ecust.edu.cn; zwqi@ecust.edu.cn

Table S1. pH for the aqueous solutions (0.01 mol/L) of MIm-PTSA mixtures with different molar ratios (20 ºC) and corresponding melting point.

entry	MIm:pTSA	pH	melting point (ºC)
1	1:0	10.13	142-143
2	3:1	7.41	39
3	4:3	6.91	9
4	1:2	1.82	16
5	0:1	1.32	103

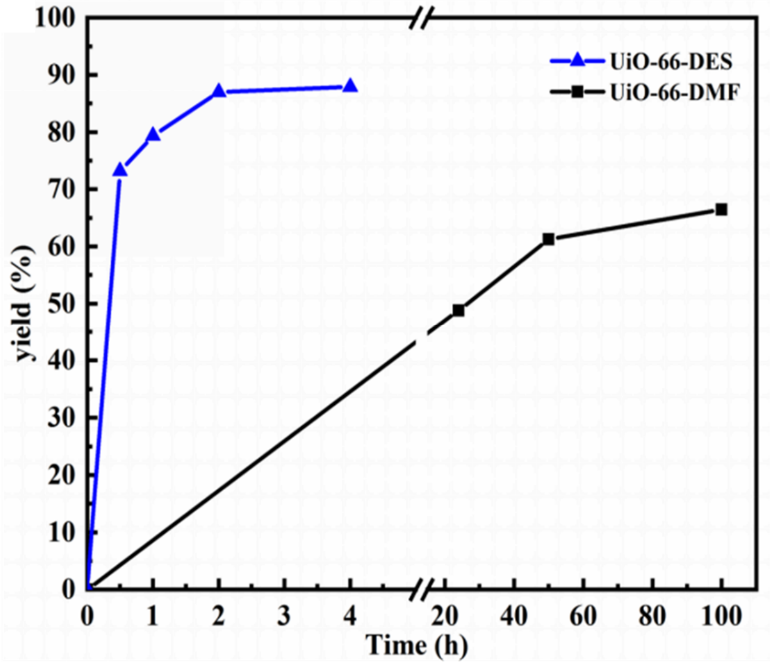

Figure S1. Formation rate of UiO-66 in DES and DMF.

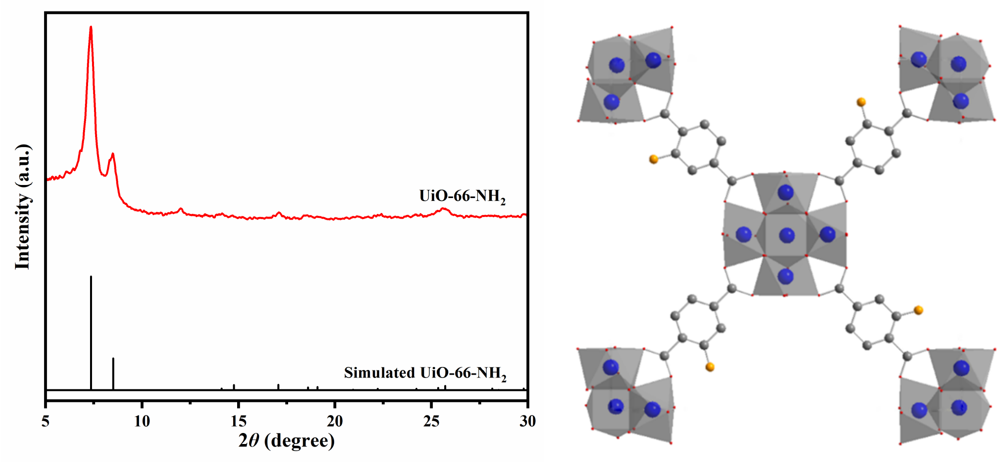

Figure S2. XRD patterns of simulated and synthesized UiO-66-NH_2, and the crystal structure of UiO-66-NH_2.

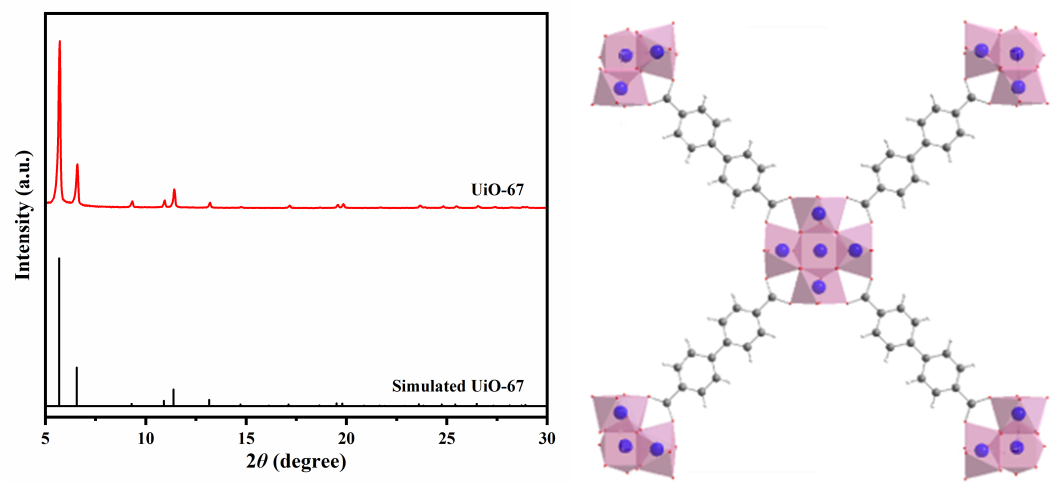

Figure S3. XRD patterns of simulated and synthesized UiO-67, and the crystal structure of UiO-67.

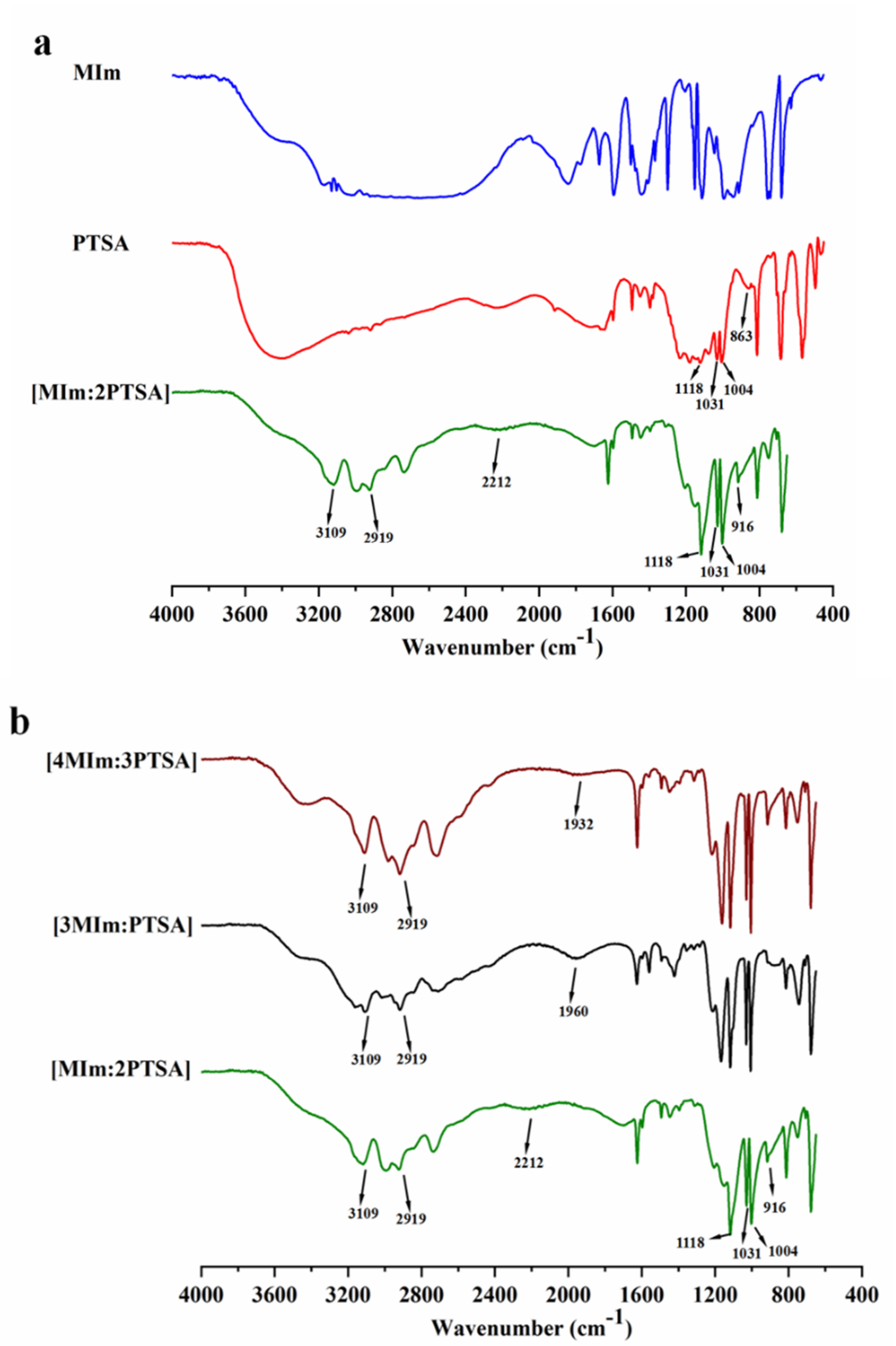

Figure S4. (a) FT-IR spectra of MIm, PTSA, and [Mim:2PTSA]; (b) FT-IR spectra of [4Mim:3PTSA], [3Mim:PTSA], and [Mim:2PTSA].

Table S2. The catalytic performance of UiO-66-DES in the acetalization of furfural and 5-hydroxymethylfurfural with methanol.

entry	Reactant	Time (h)	Conversion (%)
1	furfural	1	78.4
2	5-hydroxymethylfurfural	1	93.1
